# Supplementary material for: Reverted HIV-1 Mutants in CD4+ T-Cells Reveal Critical Residues in the Polar Region of Viral Envelope Glycoprotein
Source: Microbiol Spectr. 2021 Dec 22;9(3):e01653-21. doi: 10.1128/spectrum.01653-21 (PMC8693918; doi:10.1128/spectrum.01653-21)
Supplement: SUPPLEMENTAL FILE 1 — Supplemental material. Download SPECTRUM01653-21_Supp_1_seq6.pdf, PDF file, 0.1 MB [file spectrum01653-21_supp_1_seq6.pdf]

## SUPPLEMENTAL MATERIAL

## MATERIALS AND METHODS

**Cell culture.** TZM-bl cells, obtained through the NIH AIDS Reagent Program (NIH-ARP, catalog number 8129), and HEK293T cell line [American Type Culture Collection (ATCC), CRL-3216] were maintained in DMEM with 10% FBS as described (1). Hut/CCR5 cell line was maintained in RPMI1640 with 10% FBS (2). Hut/CCR5 cell line is derived from Hut78 $CD4^+$ T-cell line with high level CCR5 expression, supporting HIV-1 infection and replication (2). All cell lines used in this study were maintained at 37°C, 5% $CO_2$ and tested negative for mycoplasma contamination using a universal mycoplasma detection kit (ATCC 30-1012K).

**HIV-1 production.** HIV-1 containing S534P mutation (M1, M3 and M4) are not able to initiate virus-cell fusion (1). To achieve single-cycle infection, WT Env or VSV-G was used to pseudotype these viruses for efficient cell entry. To generate M1/Env, M3/Env and M4/Env viruses, HEK293T cells were co-transfected with M1, M3 or M4 proviral DNA plasmids and pIIIenv, a plasmid expressing WT Env of HIV-$1_{NL4-3}$, using polyethylenimine as described (1). For HIV-1 production of M1/VSV-G, M3/VSV-G and M4/VSV-G, HEK293T cells were co-transfected with the mutant proviral DNA plasmids and pMD2.G that expresses VSV-G protein. pMD2.G was a gift from Didier Trono (Addgene plasmid number 12259). Culture medium was changed at 6 h post-transfection, and cell supernatants were collected at 48 h post-transfection as described (1).

**HIV-1 infection and passage in cells.** Hut/CCR5 cells were infected with WT replication-competent HIV-$1_{NL4-3}$, single-cycle WT Env- or VSV-G-pseudotyped mutant viruses as described

(1, 3). At 2 h post-infection, cells were washed 3 times with phosphate buffered saline (PBS) to remove extra-cellular free viruses. Fresh media were added every 2-3 days. For cells infected with WT or Env-pseudotyped mutant viruses, cell supernatants were collected at 7 dpi. For cells infected with VSV-G-pseudotyped viruses, since syncytia appear later than WT HIV-1, culture media were collected at 13 dpi. Fresh Hut/CCR5 cells were added to the culture at 11 dpi to facilitate virus recovery.

**HIV-1 p24 quantification and kinetics of HIV-1 infection in Hut/CCR5 cells.** HIV-1 p24 levels in replication-competent viral stocks were quantified by an enzyme-linked immunosorbent assay using anti-p24-coated plates (AIDS and Cancer Virus Program, NCI-Frederick, MD) as described (4). HIV-1$_{NL4-3}$ PR-mutant M1, M3, or M4 pseudotyped either with WT Env or VSV-G were used to infect $4 \times 10^5$ Hut/CCR5 cells with equal amounts of viruses (400 ng p24) for 2 h. After infection cells were washed three times with PBS and then cultured in complete medium for the indicated times. WT HIV-1$_{NL4-3}$ was used as a positive control in the infection assay. The supernatants containing HIV-1 were collected every two days from 3 to 15 dpi for measuring viral infectivity. To maintain the viability of WT HIV-1$_{NL4-3}$ infected cells, $1 \times 10^6$ fresh Hut/CCR5 cells were also supplied to the infected cells. To quantify viral infectivity, viruses equivalent to 0.2 ng p24 were used to infect $5 \times 10^4$ TZM-bl cells in 24-well plates for 2 h and then washed out. The infected TZM-bl cells were cultured for 2 days and lysed for the luciferase assay as described (1). Cellular protein concentrations were quantified using a bicinchoninic acid assay (Pierce) and all luciferase results were normalized based on total protein input.

**Cloning and sequencing of HIV-1 *env* gene.** The HIV-1 PR mutants (M1, M3 and M4) have been reported in our recent study (1). To amplify the *env* gene of infectious viruses recovered from WT Env- or VSV-G-pseudotyped mutant viruses, Hut/CCR5 cells were infected with the recovered viruses for 3 days, the cells were collected and cellular DNAs were extracted with Blood & Tissue Kit (QIAGEN, catalog number 69504). After PCR amplification using high-fidelity DNA polymerase (Invitrogen, catalog number 12532-016), the full-length *env* cDNA were inserted into a pRK vector as described (5). After transformation into Stbl2 competent cells, colonies were randomly picked and cultured for plasmid DNA extraction. After digestion with *Sal*I and *Not*I endonucleases (New England BioLabs) to confirm the *env* cDNA insertion, 6 clones from each recovered virus were selected for Sanger DNA sequencing.

## SUPPLEMENTAL REFERENCES


1. Lu W, Chen S, Yu J, Behrens R, Wiggins J, Sherer N, Liu SL, Xiong Y, Xiang SH, Wu L. 2019. The Polar Region of the HIV-1 Envelope Protein Determines Viral Fusion and Infectivity by Stabilizing the gp120-gp41 Association. J Virol 93:e02128-18.

2. Wu L, Martin TD, Vazeux R, Unutmaz D, KewalRamani VN. 2002. Functional evaluation of DC-SIGN monoclonal antibodies reveals DC-SIGN interactions with ICAM-3 do not promote human immunodeficiency virus type 1 transmission. J Virol 76:5905-14.

3. Lu W, Tirumuru N, St Gelais C, Koneru PC, Liu C, Kvaratskhelia M, He C, Wu L. 2018. N(6)-Methyladenosine-binding proteins suppress HIV-1 infectivity and viral production. J Biol Chem 293:12992-13005.

4. Tirumuru N, Zhao BS, Lu W, Lu Z, He C, Wu L. 2016. N(6)-methyladenosine of HIV-1 RNA regulates viral infection and HIV-1 Gag protein expression. eLife 5:e15528.

5. Chen S, Bonifati S, Qin Z, St Gelais C, Kodigepalli KM, Barrett BS, Kim SH, Antonucci JM, Ladner KJ, Buzovetsky O, Knecht KM, Xiong Y, Yount JS, Guttridge DC, Santiago ML, Wu L. 2018. SAMHD1 suppresses innate immune responses to viral infections and inflammatory stimuli by inhibiting the NF-kappaB and interferon pathways. Proc Natl Acad Sci U S A 115:E3798-E3807.
